# Repurposing Immunomodulatory Imide Drugs (IMiDs) in Neuropsychiatric and Neurodegenerative Disorders

**DOI:** 10.3389/fnins.2021.656921

**Published:** 2021-03-29

**Authors:** Yoo Jin Jung, David Tweedie, Michael T. Scerba, Dong Seok Kim, Maria Francesca Palmas, Augusta Pisanu, Anna R. Carta, Nigel H. Greig

**Affiliations:** ^1^Drug Design & Development Section, Translational Gerontology Branch, Intramural Research Program, National Institute on Aging, National Institutes of Health, Baltimore, MD, United States; ^2^Stanford Neurosciences Interdepartmental Program, Stanford University School of Medicine, Stanford, CA, United States; ^3^AevisBio, Inc., Gaithersburg, MD, United States; ^4^Aevis Bio, Inc., Daejeon, South Korea; ^5^Department of Biomedical Sciences, University of Cagliari, Cagliari, Italy; ^6^National Research Council, Institute of Neuroscience, Cagliari, Italy

**Keywords:** IMiDs^®^, neurodegenarative diseases, neuropsychaitric disorders, neuroinflammation, thalidomide, pomalidomide

## Abstract

Neuroinflammation represents a common trait in the pathology and progression of the major psychiatric and neurodegenerative disorders. Neuropsychiatric disorders have emerged as a global crisis, affecting 1 in 4 people, while neurological disorders are the second leading cause of death in the elderly population worldwide (WHO, 2001; GBD 2016 Neurology Collaborators, 2019). However, there remains an immense deficit in availability of effective drug treatments for most neurological disorders. In fact, for disorders such as depression, placebos and behavioral therapies have equal effectiveness as antidepressants. For neurodegenerative diseases such as Parkinson’s disease and Alzheimer’s disease, drugs that can prevent, slow, or cure the disease have yet to be found. Several non-traditional avenues of drug target identification have emerged with ongoing neurological disease research to meet the need for novel and efficacious treatments. Of these novel avenues is that of neuroinflammation, which has been found to be involved in the progression and pathology of many of the leading neurological disorders. Neuroinflammation is characterized by glial inflammatory factors in certain stages of neurological disorders. Although the meta-analyses have provided evidence of genetic/proteomic upregulation of inflammatory factors in certain stages of neurological disorders. Although the mechanisms underpinning the connections between neuroinflammation and neurological disorders are unclear, and meta-analysis results have shown high sensitivity to factors such as disorder severity and sample type, there is significant evidence of neuroinflammation associations across neurological disorders. In this review, we summarize the role of neuroinflammation in psychiatric disorders such as major depressive disorder, generalized anxiety disorder, post-traumatic stress disorder, and bipolar disorder, as well as in neurodegenerative disorders, such as Parkinson’s disease and Alzheimer’s disease, and introduce current research on the potential of immunomodulatory imide drugs (IMiDs) as a new treatment strategy for these disorders.

## Introduction

Chronic neuroinflammation is a common feature across numerous neurological disorders, including neurodegenerative diseases, myelin disorders, and several psychiatric disorders (Goldsmith et al., 2016; Han et al., 2019; Jung et al., 2019; Yuan et al., 2019). Whereas it is a recognized pivotal player in the progression of neurodegeneration in Parkinson’s disease (PD) and in Alzheimer disease (AD), neuroinflammation also appears to be heavily involved in the pathophysiology of psychiatric disorders, including major depressive disorder (MDD) and bipolar disorder (BD) (Beurel et al., 2020). Therefore, although the origin of neuroinflammation may vary depending on the neurological illness and is often poorly understood, modulation of the inflammatory response may represent a wide target to mitigate neurological disorders. Neuroinflammation is chiefly mediated by cytokine-releasing reactive microglia within the CNS (Clark et al., 2010). Cytokines are secreted and regulated in cascades, acting to increase the downstream production of other cytokines and to amplify the inflammatory response (Kronfol and Remick, 2000). Among the many inflammatory cytokines that play a part in propagating neuroinflammation, tumor necrosis factor-alpha (TNF-α) acts as a master regulator of downstream inflammatory pathways (Parameswaran and Patial, 2010). Importantly, central cytokine levels can be affected by peripheral levels, as the blood-brain barrier (BBB) may be disrupted or become more permeable in the progression of neurological disorders (Benatti et al., 2016; Menard et al., 2017; Sweeney et al., 2018).

Based on the pivotal role of neuroinflammation in neurological disorders, anti-inflammatory and immunomodulatory agents, as well as anti-TNF agents, have been considered and have shown potential to prevent or alleviate symptoms of psychiatric or neurodegenerative disorders (Martinez and Peplow, 2018; Beurel et al., 2020). In the quest of identifying candidate agents with immunomodulatory and neuroprotective activity, the repositioning of immunomodulatory imide drugs (IMiDs) has raised great interest in the last decade. In this review article, we overview IMiDs, the source of a series of close analogs with potent anti-inflammatory activity that have proved hugely valuable in the treatment of multiple myeloma, addressing both their promise as well as Achilles heel. We summarize evidence of inflammation and elevated cytokine levels in neuropsychiatric and neurodegenerative disorders, with an emphasis on TNF-α. We then discuss current preclinical and clinical evidence of potential beneficial effects of IMiDs in neurodegenerative disorders, and propose IMiDs as a prospective new treatment strategy for neurodegenerative and neuropsychiatric disorders. Although IMiDs have yet to be studied in the context of neuropsychiatric disorders, they may offer a revolutionary therapy in light of the newly recognized role of inflammation in these illnesses.

## Immunomodulatory Imide Drugs (IMiDs)

**FIGURE 1:**
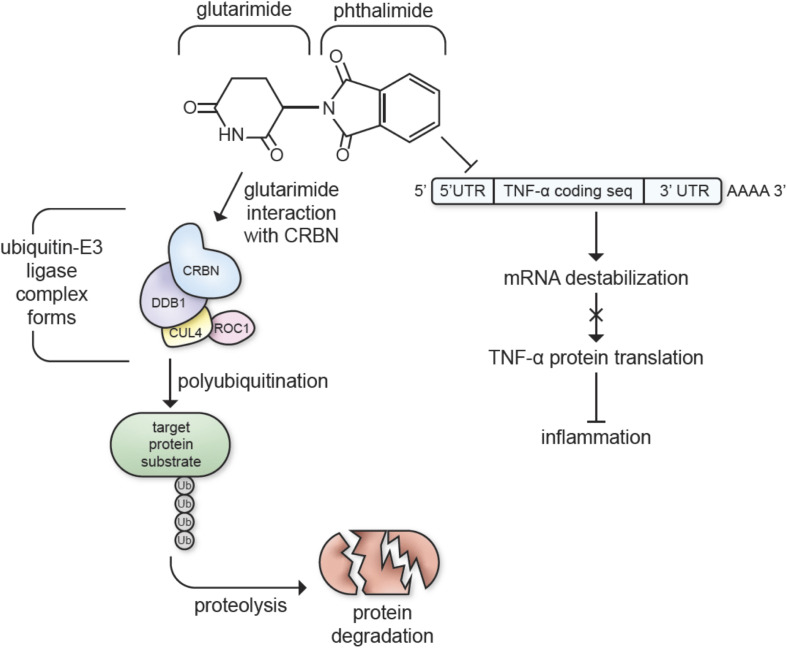
Immunomodulatory imide drugs interactions with cereblon (CRBN) and TNF-α mRNA. IMiDs destabilize the 3′-untranslated region (3′-UTR) of TNF-α mRNA, inhibiting TNF-α protein synthesis and inhibiting inflammatory pathways (Moreira, 1993; Zhu et al., 2003). The glutarimide moiety of the thalidomide backbone catalyzes E3 ubiquitin ligase complex formation, targeting proteins for proteolysis (Chamberlain et al., 2014; Fischer et al., 2014; Petzold et al., 2016; Steinebach et al., 2018).

Immunomodulatory imide drugs are analogs of the drug thalidomide that possess pleiotropic anti-myeloma actions. These comprise of anti-proliferative, anti-angiogenic, immune-modulatory and, in particular, potent anti-inflammatory effects, with the latter due to their ability to inhibit the production of the proinflammatory cytokine TNF-α. TNF-α plays a central role in microglial activation and in the propagation of the inflammatory response that, when dysregulated, may lead to neurotoxicity or neuronal dysfunction (Sriram and O’Callaghan, 2007; Baker et al., 2011; Figure 1).

Aside from their TNF-α inhibiting properties, most IMiDs, excluding apremilast and N-adamantyl phthalimidine, bind cereblon (CRBN), a protein involved with cellular protein degradation that is critically engaged in many of thalidomide and analog’s actions (Mendy et al., 2012; Chamberlain et al., 2014; Ito and Handa, 2016; Millrine and Kishimoto, 2017; Shi and Chen, 2017; Hsueh et al., 2021; Figure 1). CRBN forms an E3 ubiquitin ligase complex with DNA damage-binding protein-1 (DDB1), Cullin 4 (Cul4A/B), and regulator of Cullins 1 (RoC1). The CRBN component of this complex targets proteins for degradation via a ubiquitin-proteasome pathway, ultimately decreasing neuronal excitotoxicity and metabolic dysfunction when used to target dysfunctional proteins in the neural environment (Shi and Chen, 2017). IMiDs are known to recruit several neo-substrates of CRBN to the E3 ubiquitin ligase complex, and this results in the diverse biological and pharmacological actions of IMiDs (Lu et al., 2015; Winter et al., 2015; Donoghue et al., 2020; Yang et al., 2020; Figure 1). In particular, this CRBN-binding capability underlies the actions of IMiDs in the treatment of multiple myeloma. Specifically, the glutarimide moiety of the thalidomide backbone binds to CRBN, which modulates the molecular environment of its substrate binding surface preference to target proteins such as the Ikaros zinc finger family proteins Ikaros (IKZF1) and Aiolos (IKZF3) (Chamberlain et al., 2014; Fischer et al., 2014; Petzold et al., 2016; Steinebach et al., 2018). IMiD treatment leads to degradation of IKZF1/3 that, in turn, leads to upregulation of IL-2, an inflammatory cytokine, stimulating T cells to attack myeloma cells (Haslett et al., 2005; Gandhi et al., 2014).

Although useful in underpinning the anticancer actions of IMiDs in multiple myeloma, their binding to CRBN unpins the Achilles’ heel of this drug class, specifically the notorious teratogenic effects. Marketed in 1957 by Chemie-Grunenthal as a non-addictive, non-toxic, non-barbiturate sedative, thalidomide was widely prescribed to treat morning sickness in pregnant women. This resulted in over 10,000 children born with a range of severe and debilitating malformations – now considered one of the biggest ever man-made medical disasters (Vargesson, 2015). Extensive recent studies have linked this to CRBN-binding and subsequent targeted interaction with and ubiquitination of the Cys2/His2-type (C2H2) zinc finger transcription factor Sal-like protein 4 (SALL4) (Donovan et al., 2018; Matyskiela et al., 2018). The degradation of SALL4 associated with IMiD-linked teratogenicity aligns with independent studies demonstrating that mutations in SALL4 result in a number of human conditions that share striking similarities to thalidomide embryopathy. These include Duane-Radial ray syndrome, Acro-Renal-Ocular syndrome and IVIC syndrome (sometimes known as Oculo-oto-radial syndrome) (Vargesson, 2019). As a consequence, thalidomide-like drugs carry a “boxed warning” in relation to teratogenicity, requiring the use of two reliable forms of birth control beginning 4 weeks prior to drug treatment and ending 4 weeks after treatment termination. This duality of IMiDs has, in part, been attributed to the racemization of IMiDs around their chiral center (the C3-carbon atom of the glutarimide ring), into a mixture of R- and S-enantiomers. It has been reported that whereas the S-enantiomer is primarily responsible for the teratogenic effects of this drug class, the R-enantiomer enables its pharmacological activity (Ríos-Tamayo et al., 2017). However, when generated and administered as a chirally pure enantiomeric form, interconversion of the enantiomers occurs under physiological conditions (chiral switching), unavoidably resulting in a racemic mixture (Mori et al., 2018). Nevertheless, it has been possible to evaluate configurationally stable forms of thalidomide and analogs, which can be achieved by replacing the acidic H atom on the chiral (α) carbon with a more stable F that is a poor leaving group. This effectively inhibits racemization, as described for 3-fluoro thalidomide (Takeuchi et al., 1999; Man et al., 2003; Lee et al., 2011; Tokunaga et al., 2017; Mori et al., 2018). *S-* and *R-* chirally stable analogs of thalidomide and analogs have shown different binding to CRBN, resulting in different downstream actions on IKZF1/3 and SALL4 (Mori et al., 2018).

Multiple crystallographic studies involving human, mouse as well as chick CRBN have characterized the means through which thalidomide and analogs interact with CRBN. This involves a shallow pocket (sometimes termed the thalidomide-binding domain) that is formed by three conserved surface tryptophan (Trp) residues on the central β-sheet relatively close to the surface of CRBN (Chamberlain et al., 2014). The glutarimide ring of thalidomide and analogs docks into grooves created by Trp383, Trp403, Trp389, and His381 side chains, and with an approximately 10-fold greater potency for the S-enantiomeric form (Mori et al., 2018). In all cases, the phthalimido portion of the compound protrudes out of the binding domain to allow interaction with neo-substrates, such as SALL4, with the R-enantiomeric form having a slight twist to mitigate steric clashes but, nevertheless, it still interacts with CRBN and generates teratogenicity, albeit at higher necessitated concentrations than those associated with the S-enantiomer (Mori et al., 2018). Thalidomide’s S-enantiomeric form fits more readily in CRBN’s binding domain with a relaxed conformation, and induces teratogenicity at an approximately 10-fold lower concentration (Asatsuma-Okumura et al., 2020).

Together with a lack of teratogenicity studies that are now required by regulatory agencies across the world, species-specific actions of thalidomide and analogs may have contributed to the thalidomide birth defect tragedy. In rodents, thalidomide-induced teratogenic effects are not observed (Fratta et al., 1965; Gemechu et al., 2018). Whereas murine CRBN is approximately 95% homologous to the human protein and readily binds thalidomide and clinical analogs, subsequent ubiquitination and degradation of neo-substrates, exemplified by IKZF1/3 and SALL4, does not occur consequent to two key amino acid differences between rodent and human CRBN (Asatsuma-Okumura et al., 2020). In this regard, human CRBN 377 glutamic acid (E377) is replaced by a valine (V), and human 388 valine (V388) by an isoleucine (I). Notably, the CRBN substitution V388I eliminates thalidomide-mediated interaction with IKZF1 as well as with protein kinase CK1α (Gemechu et al., 2018; Asatsuma-Okumura et al., 2020). Furthermore, a recent study using human induced pluripotent stem cells demonstrated that CRBN V388I mutation abolished thalidomide-induced degradation of SALL4, and that this involved a specific interaction with SALL4 416 glycine (G416) whose mutation (G416A), likewise abrogated SALL4 breakdown (Belair et al., 2020). Such studies provide an insight as to how select actions of thalidomide and clinical analogs are mediated via CRBN, but have yet to fully explain the development of teratogenicity whose resistance in rodents remains enigmatic (Vargesson, 2015, 2019; Asatsuma-Okumura et al., 2020).

A non-C2H2 zinc finger-type protein that potentially is also a thalidomide-mediated CRBN neo-substrate is the tumor protein p63, which is a member of the p53 family of transcription factors and has pleiotropic function that include roles in cell proliferation, survival, apoptosis, differentiation, development, tumorigenesis, senescence and aging (Soares and Zhou, 2018). In excess of 10 isomers of p63 have been isolated and two key ones, ΔNp63α and TAp63α, have been identified as thalidomide-dependent CRBN neo-substrates in zebrafish – with ΔNp63α being essential for limb development, whereas TAp63α appears critical for cochlea development and hearing (Asatsuma-Okumura et al., 2019). The expression of both p63 forms was reduced on thalidomide exposure (Asatsuma-Okumura et al., 2020).

The anti-inflammatory mechanisms of thalidomide and its derivatives does not appear to require binding to CRBN. Studies investigating the activity of IMiDs in relation to CRBN binding and anti-inflammatory effects have demonstrated the anti-inflammatory activity of some IMiDs in the absence of CRBN binding. A good example of this is the recently developed adamantyl thalidomide compound, N-adamantyl phthalimidine (NAP), shown to mitigate LPS-induced elevations in proinflammatory cytokines in cellular and animal models and provide anti-inflammatory effects in models of TBI without binding to CRBN (Hsueh et al., 2021). In this regard, the three-dimensional cage-like structure of its adamantyl-like moiety that replaces the relatively flat glutarimide ring is too large to fit into the thalidomide binding domain of CRBN (Peach et al., 2020). As would be predicted in its lack of binding affinity to CRBN, NAP exposure does not result in SALL4 degradation in cellular studies (Hsueh et al., 2021), and studies on p63 and *in vivo* models of teratogenicity are awaited with interest.

As discussed, currently marketed IMiDs have prescription limitations and a boxed warning for females within child-bearing age. Given that anxiety and mood disorders disproportionately affect women over men and are one of the greatest causes of global disease burden in women, the development of IMiDs that do not bind to CRBN is highly warranted and will greatly enhance the repurposing capabilities of IMiDs (Nolen-Hoeksema, 2001; Slavich and Sacher, 2019). On this basis, NAP has been recently proposed as a promising IMiD for targeting neuroinflammation in neurological conditions of female patients within child-bearing age that warrants further investigation and, importantly, toxicological evaluation with a focus on teratogenicity (Hsueh et al., 2021).

**FIGURE 2:**
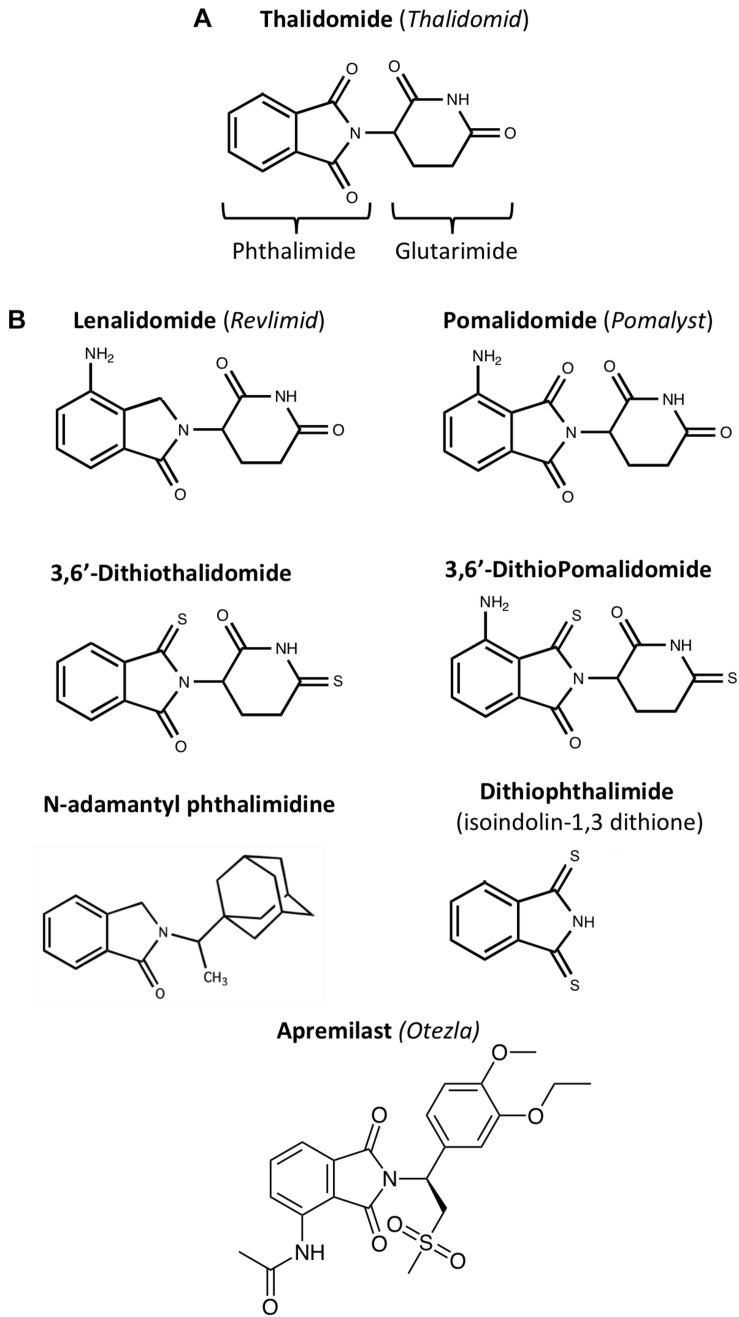
Chemical structures of IMiDs: **(A)** Thalidomide comprises of conjoined phthalimide and glutarimide moieties – with a chiral center. **(B)** Clinically approved (lenalidomide, pomalidomide and apremilast) and experimental IMiDs: IMiDs can be structurally altered to enhance function, increase bioavailability, and reduce adverse side effects (Zhu et al., 2003; Luo et al., 2011, 2018). For instance, the CNS MPO Score of thionated Pomalidomide, 3,6′-Dithiopomalidomide (3,6′-DP), is 5.5, making it higher than that of Pomalidomide, which has a score of 4.8; CNS MPO Scores predict drug BBB permeability calculated by Chemicalize using BBB penetration factors such as molecular weight and topological polar surface area (see Table 1).

As IMiDs are multi-potent, several IMiDs - such as thalidomide (*Thalidomid*), lenalidomide (*Revlimid*), pomalidomide (*Pomalyst*), and Apremilast (*Otezla*) - have been repurposed as drug treatments for diseases such as multiple myeloma and psoriatic arthritis, and more recently, Kaposi Sarcoma (Bristol Myers Squibb, 2020). Moreover, thalidomide remains a treatment choice for erythema nodosum leprosum (ENL), an inflammatory complication of leprosy, which was first described in Sheskin (1965) and FDA approved in 1998 (Melchert and List, 2007; Asatsuma-Okumura et al., 2020). Preclinical studies of FDA approved IMiDs, as well as novel IMiDs such as 3,6′-dithiopomalidomide (3,6′-DP) and adamantyl thalidomide derivatives, support repurposing of IMiDs as therapeutics for neurological diseases with inflammatory components, such as AD, PD, stroke, traumatic brain injury and multiple sclerosis (Russo et al., 2012; He et al., 2013; Yoon et al., 2013; Eitan et al., 2015; Wang et al., 2016; Boi et al., 2019; Casu et al., 2020; Lin et al., 2020; Hsueh et al., 2021; Figure 2). Compared with classical immunosuppressants and TNF-α-targeting drugs such as Etanercept and Infliximab, the pharmacokinetic features of IMiDs offer several advantages that make them more suitable for treating chronic neurological disorders. Notably, they are orally deliverable, highly bioavailable, and permeable to the BBB. In contrast, Etanercept and Infliximab, which are macromolecules, rely on subcutaneous and intravenous administration, respectively. Despite their high specificity, limited off-target toxicity and relatively long serum half-life, in comparison to small molecule drugs, their uptake across the BBB is minimal. They routinely attain brain levels 0.2% or less than concomitant plasma levels, posing a substantial challenge to drug development and treatment of neuroinflammation (Pardridge, 2012; Karaoglu Hanzatian et al., 2018). Elegant preclinical and clinical studies by Tobinick (2010, 2016, 2018) have utilized perispinal injection together with Trendelenburg positioning (inverting the subject for a few minutes immediately post-drug administration) to augment macromolecule central nervous system (CNS) delivery. Nonetheless, the availability of brain penetrant small molecular weight orally bioavailable drugs would likely have wider utility.

In this regard, most IMiDs have high CNS MPO (multiparameter optimization) scores (Table 1), which quantify predictions of drug BBB permeability based on factors such as molecular weight, hydrogen bond donors and acceptors, and topological polar surface area (Wager et al., 2010; Jung et al., 2019). A high BBB permeability allows IMiDs to be delivered to the brain at clinically relevant oral doses. IMiDs also follow the Lipinski rule of five, predicting their successful delivery to their drug target under physiological conditions (Banks and Greig, 2019; Table 1). Confirming *in silico* data, i*n vivo* pharmacokinetic studies of IMiDs have shown that IMiDs such as 3,6′-DP readily enter the brain from plasma (with brain/plasma concentration ratios of approximately 0.85, i.e., close to unity; Lin et al., 2020).

**TABLE 1:** Calculated CNS MPO score, log *P*-value and related factors that impact drug pharmacokinetics and brain entry under physiological conditions: Molecular weight, Log *P*-value, the quantity of hydrogen bond donors and acceptors, the topological polar surface area value, and the pKa were computed with Chemicalize for several clinically approved and available IMiDs as well as for the recent analogs 3,6′-dithio-pomalidomide (3,6′-DP), -thalidomide (3,6′-DTT) and the adamantyl moiety containing agent N-adamantyl pththalmidine.

**Compound**	**Molecular weigh**	**Log P**	**Topological polar**	**Number of hydrogen**	**Number of hydrogen**	Most basic center acidity*	**CNS MPO**
**Compound**	**(g/mol)**		**surface area (Å2)**	**bond donors**	**bond acceptors**	**(calculated pK_a_)**	**score**
Thalidomide (*Thalidomid*)	258.2	0.02	83.55	1	4	11.59**	4.8
Lenalidomide (*Revlimid*)	259.3	–0.71	92.50	2	4	2.31	5.4
Pomalidomide (*Pomalyst*)	273.2	–0.16	109.57	2	5	1.56	4.8
Apremilast (*Otezla*)	460.5	1.31	119.08	1	7	12.98**	3.1
3,6′-Dithiopomalidomide (3,6′-DP)	305.4	0.97	75.43	2	3	2.33	5.5
3,6′-Dithiothalidomide (3,6′-DTT)	290.4	1.80	49.41	1	2	9.8**	4.9
N-adamantyl phthalimidine (NAP)	295.4	3.86	20.31	0	1	–1.04	3.7
Dithiophthalimide	179.3	2.47	12.03	1	0	14.31**	3.6

Such IMiDs largely are in accord with the Lipinski rule of five, which predicts the likelihood of a drug being delivered to its target under human physiological conditions based on its physical properties (Banks and Greig, 2019). A CNS MPO Score, that provides an estimate of a drug’s BBB permeability, was computed using parameters described by Wager et al. (2010), with a greater score value associating with a higher drug BBB permeability. *Obtained from Chemicalize as the calculated “Strongest Basic pKa” and presumably as if the parent molecule were to function as a base. When such a value was not computed or available from the software, the Strongest Acidic pKa value was utilized instead. **Indicates the Strongest Acidic pKa value; subsequently used for MPO calculation.

Furthermore, Pomalidomide, one of the most recently FDA-approved and potent IMiDs currently on the drug market, has lately been reported to effectively suppress inflammatory factors and inflammation-induced neuronal injury in cell and animal models of neurological diseases and cellular stress (Wang et al., 2016; Tsai et al., 2018, 2019). In this regard, pomalidomide has demonstrated TNF-α inhibitory action of up to 50,000-fold greater than that of thalidomide (Mahony et al., 2013; Wang et al., 2016) and has a favorable BBB permeability in mice, achieving a brain/plasma concentration ratio of 0.71 (Tsai et al., 2019). Additions and substitutions on the chemical structure of pomalidomide have been performed in recent years to augment drug efficiency and have yielded novel analogs with enhanced biological activity, exemplified by thionated 3,6′-DP. Recent studies have demonstrated the efficacy of 3,6′-DP in rodent TBI models at one fifth (i.e., 20%) of the dose required by pomalidomide; with 3,6′-DP providing more potent and broader inhibitory effects on inflammatory cytokines and cellular stress molecules such as nitric oxide and COX-2 (Lin et al., 2020).

## Neuroinflammation in Neurological Disorders

Neuroinflammation is initiated in response to stimuli such as cranial impact or pathogen infiltration, by activation of microglia, the immune cells of the brain. Although neuroinflammation serves to repair and reinstate the neural environment to normal conditions and ensure homeostasis, an exaggerated neuroinflammatory response can result in the chronic production and release of an elevated amount of proinflammatory cytokines by microglia, triggering a self-fueling loop that can perpetuate neuroinflammation and contribute to neuronal death or neuronal dysfunction (Frankola et al., 2011; Heneka et al., 2015).

Among proinflammatory cytokines produced by microglia, systemic expression of the inflammatory cytokine TNF-α holds important physiological functions in CNS development and homeostasis, as well as in the homeostasis of peripheral systems, such as the cardiovascular system (Haider and Knöfler, 2009; Mizrahi and Askenasy, 2014; Urschel and Cicha, 2015). In the neuronal environment, physiological levels of TNF-α regulate synaptic connectivity and dendritic pruning in the context of neurodevelopment and CNS homeostasis (Gilmore et al., 2004; Kaneko et al., 2008; Lewitus et al., 2016; Liu et al., 2017; Yee et al., 2017). Moreover, TNF-α acts as a neuromodulator by regulating neurotransmission. For example, TNF-α regulates AMPA receptor expression that, in large part, controls the neuronal excitability upon glutamatergic stimulation, and, more generally, the glutamatergic mechanisms underlying synaptic plasticity (Beattie et al., 2002; Stellwagen and Malenka, 2006). Glia-released TNF-α has been observed to enhance synaptic neurotransmission via AMPA receptor trafficking, whereas blocking TNF-α has been shown to have the opposite effect. Although TNF-α is widely considered to have no substantial effect on long-term potentiation (LTP) or long-term depression (LTD), TNF-α expression does alter synaptic scaling in the hippocampus and striatum to ensure the homeostasis of neuronal activity and to prevent hyper or hypoactivity (Liu et al., 2017; Rizzo et al., 2018).

In the context of injury, TNF-α is a potent activator of the immune system, and a pivotal trigger of inflammation pathways, as a protective measure against infection, viral attack and neurotoxins (Kraft et al., 2009). Although inflammation is an essential healing response, the production of TNF-α together with other proinflammatory factors can be detrimental when dysregulated (McCoy and Tansey, 2008). Extended, excessive TNF-α expression has been associated with chronic inflammation and gliosis, glutamatergic toxicity-induced apoptosis, and synaptic loss (Clark et al., 2010). Many neurological disorders appear to share a neuroinflammatory component, and chronic TNF-α expression has been observed in neurodegenerative disorders such as multiple sclerosis, amyotrophic lateral sclerosis, PD, AD, ischemia, as well as in various forms of dementia (Lee Y. J. et al., 2010; Decourt et al., 2016; Hu et al., 2019). Similarly, TNF-α increases have been observed in several neuropsychiatric disorders, including MDD, BD, and schizophrenia, and potentially contributes to the neuropathology of these disorders (Bandelow et al., 2017; Kopschina Feltes et al., 2017).

As the excessive, prolonged or chronic release of TNF-α and related inflammatory responses are neurotoxic, they themselves can become self-amplifying and make the neural environment more prone to further genomic and proteomic dysfunction and dysregulation, TNF-α may therefore represent an ideal drug target for mitigating neurological conditions with underpinning inflammatory components, such as neuropsychiatric and neurodegenerative disorders (Husain et al., 2017; Ramos-Cejudo et al., 2018; Köhler-Forsberg et al., 2019).

### Neuropsychiatric Disorders

There is an increasing need for effective drug development for neuropsychiatric disorders, which continue to be an enormous health burden worldwide, especially as patient responsiveness to existing drugs, particularly antidepressants, remains as low as 30-50% (Dowlati et al., 2010). Meta-analyses on FDA-approved antidepressants have revealed that existing antidepressants have low modulatory effects on neurotransmitter dysregulation underlying depression and anxiety disorders. This is evident on a macro level, with 30% of MDD patients and nearly half of BD patients experiencing refractory depressive episodes in response to current antidepressants (Tondo et al., 2014; Hashimoto, 2019). Placebos and cognitive therapy methods (i.e., psychotherapy, exercise) have been reported to be just as effective and safer than antidepressants for MDD and depressive phases of BD (Kirsch, 2019). Furthermore, antidepressants have been reported to be poorly effective in preventing suicidal behavior in patients under the age of 25 (Grunebaum and Mann, 2007), stressing the need for more effective drug treatments. On the other hand, long-term treatment of psychiatric disorders with antipsychotic drugs is often accompanied by severe adverse effects, such as movement or metabolic disorders (Dold et al., 2015; Hirsch et al., 2017), indicating yet a further need for more effective and less harmful drugs for these disorders.

During the last decade, the role of inflammation in neuropsychiatric disorders has gained increasing support (Miller and Raison, 2016; Jeon et al., 2019; Momtazmanesh et al., 2019; Felger and Miller, 2020). Meta-analyses of systemic cytokine concentration in patients with acute bipolar mania and MDD demonstrated significantly increased serum TNF and IL-6 compared with controls (Goldsmith et al., 2016). Similarly, a cross-disorder study on meta-analyses of 8 different psychiatric disorders, including MDD, BD, and Post-traumatic stress disorder (PTSD), showed significant changes in several inflammatory markers in the blood or cerebrospinal fluid (CSF), with factor clustering based on disorder type and stage (Yuan et al., 2019). Within the CNS, neuroinflammation and reactive glial cells have often been reported in neuropsychiatric conditions (Uranova et al., 2004; Brietzke and Kapczinski, 2008; Rege and Hodgkinson, 2013). Peripheral cytokines can cross the BBB and interact with central immune cells to support neuroinflammatory responses. The cytokine passage rate of the BBB has been associated with symptoms of psychiatric disorders such as MDD, suggesting that monitoring and targeting elevated cytokines may be a viable option for psychiatric disorder intervention (Yarlagadda et al., 2009). Within the CNS, cytokines may affect the function of neuronal circuitry involved in psychiatric symptoms, leading to changes in mood and behaviors such as sleep, reward, motivation, and to depressive and cognitive symptoms (Mössner et al., 1998; Yarlagadda et al., 2009; Donegan et al., 2014; Watkins et al., 2014). It is interesting to note that stress and trauma, which are associated with the onset of psychiatric disorders such as BD and PTSD, increase the release of cytokines and cortisol. In this regard, inflammatory cytokines can interact with the hypothalamus, pituitary, and adrenocortical (HPA) axis, altering neurotransmitter and hormone release by the neuroendocrine system (Kim et al., 2007).

#### Major Depressive Disorder

Major depressive disorder is highly associated with a neuroinflammatory condition, and inflammation has been classically suggested to play a role in the pathophysiology of this disorder (Smith, 1991; Beurel et al., 2020). Meta-analyses have shown an upregulation of several inflammatory factors, including TNF-α, IL-6, IL-1β, IL-10, and C-reactive protein, in the context of MDD (Howren et al., 2009; Haapakoski et al., 2015). Several meta-analyses of cross-sectional studies have confirmed the increase in circulating inflammatory cytokines in MDD patients (Howren et al., 2009; Dowlati et al., 2010; Haapakoski et al., 2015; Goldsmith et al., 2016), while some longitudinal studies have suggested that elevated cytokine levels are observed before the onset of depressive symptoms and may be directly involved in depression pathophysiology (Kohler et al., 2016; Kopschina Feltes et al., 2017). Some genetic variants of IL-1β have been associated with decreased function of the amygdala and anterior cingulate cortex, which can lead to difficulties in emotional processing and worse outcome for MDD patients (Baune et al., 2010). In patients affected by major depressive disorder, signs of microglial activation such as increased translocator protein (TSPO) volume, a positron emission tomography (PET) scan marker of microglial activation (previously referred to as peripheral benzodiazepine receptors (PBR)), have been reported in comparison with healthy controls (Setiawan et al., 2015). The positive correlation between depressive episode severity and TSPO volume indicate that microglia activation and neuroinflammation may contribute to depression severity (Setiawan et al., 2015). Additionally, the involvement of neuroinflammation in MDD is supported by correlative observation studies on inflammation-related conditions (epitomized by rheumatoid arthritis and atherosclerosis) in relation to depression, and the high prevalence of depression in post-menopausal women who produce less estrogen, which has anti-inflammatory properties (Bruce-Keller et al., 2000).

Interestingly, several antidepressants seem to have anti-inflammatory effects, which might contribute to their effectiveness; for instance, fluoxetine (Prozac) and citalopram (Celexa), the top-most prescribed SSRI for depression, have been observed to decrease TNF-α levels in patients, and have been successful in treating inflammatory conditions such as rheumatoid arthritis in preclinical studies (Sacre et al., 2010; Almeida et al., 2020). Of note, a link has been found between inflammatory gene variants and antidepressant resistance. Carriers of an IL-1β allele that causes reduced IL-1β levels are more resistant to antidepressants, supporting the notion that antidepressants reduce depressive symptoms partially via inflammation modulation (Bufalino et al., 2013). Accordingly, some anti-inflammatory drugs, such as NSAIDs, have been shown to decrease depressive symptoms in preclinical studies and clinical trials (Kopschina Feltes et al., 2017). Minocycline, a tetracycline antibiotic capable of lowering microglial activation and TSPO volume in rodents, has also been shown to attenuate rodent behaviors indicative of depression (Henry et al., 2008). The anti-TNF-α drugs etanercept, adalimumab, infliximab and tocilizumab have produced a significant reduction in depressive symptoms in randomized controlled trials. This effect was not related to sex, age, or study duration, indicating a causal relationship between inflammation and MDD and the possibility for MDD treatment using inflammation-targeting compounds (Kappelmann et al., 2018). In a clinical trial, treatment of osteoarthritis patients, who are 2-3 times more prone to depression than age-matched controls, with celecoxib or ibuprofen and naproxen induced an improvement of depressive symptoms (Iyengar et al., 2013).

Additionally, natural anti-inflammatory agents such as curcumin and fish oil have shown promising results in preclinical models of MDD, by decreasing nuclear factor-kappa B (NF-κB) signaling and TNF-α production (Lichtman et al., 2008; Eng et al., 2011; Smith et al., 2011; Burhani and Rasenick, 2017; Lopresti, 2017; Maki et al., 2018; Liu T. et al., 2019; Nerurkar et al., 2019). A recent study showed attenuation of anxiety and depressive behavior and inflammation in mouse models of chronic stress through treatment with probiotics and polyphenol-rich prebiotics, or synbiotics (Westfall et al., 2021). Synbiotic-derived metabolites appear to combat inflammation by decreasing inflammasome pathway activation and immune cell recruitment to the brain and resetting peripheral T cell ratios (Westfall et al., 2021). These results offer insight on methods for decreasing peripheral inflammation through control of the gut-brain axis to consequently enhance mood and combat stress-related mood disorders.

**FIGURE 3:**
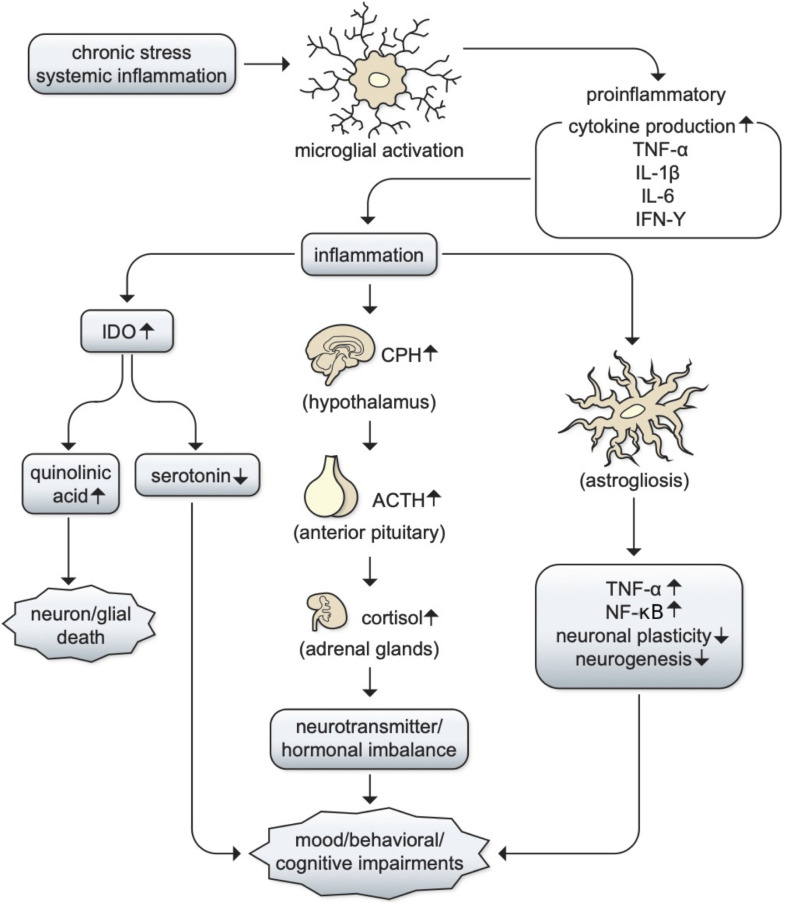
The potential role of inflammation in MDD. The relationships between inflammation and depression are still unclear, but there are two main mechanisms through which inflammation can contribute to depression: chronic stress or systemic inflammation can lead to microglial activation, which can lead to the production of proinflammatory cytokines such as TNF-α, IL-1β, IL-6, and IFN-γ. This leads to the propagation of inflammatory pathways, which can (1) activate the hypothalamic–pituitary adrenal (HPA) axis to produce cortisol and cause an imbalance in serotonin, norepinephrine, and epinephrine; (2) increase in the production of indoleamine-2,3-dioxygenase (IDO), resulting in serotonin depletion and increased quinolinic acid and contributing to cell death (Kopschina Feltes et al., 2017). It is likely that both pathways could underlie MDD progression and neurotransmitter imbalances that lead to various comorbid symptoms such as mood, behavioral, or cognitive impairments such as sleep, concentration, and cognition (Mössner et al., 1998; Donegan et al., 2014; Watkins et al., 2014).

The relationship between inflammation and depression remains largely unclear, but there are two main mechanisms through which inflammation can contribute to depression: (1) an imbalance in serotonin, norepinephrine, and epinephrine production following hypothalamic–pituitary adrenal (HPA) axis activation; (2) increased activity of the inflammation by-product indoleamine-2,3-dioxygenase (IDO), resulting in serotonin depletion and increases in quinolinic acid (Kopschina Feltes et al., 2017). MDD patients have reported depression ratings directly correlating with levels of cortisol, the resulting product of HPA axis activation (Gibbons and McHugh, 1962). Moreover, abnormal cortisol responses have been associated with anxiety and depression symptoms following stress induction in non-patients, further supporting the former mechanism (Carpenter and Bunney, 1971; Pearson Murphy, 1991; Fiksdal et al., 2019). Additionally, chronic stress-induced inflammation has been shown to lead to increased levels of kynurenine metabolites, which are catabolized by IDO1 and are associated with alterations in brain regions involved in emotional regulation, supporting the latter mechanism (Kim and Won, 2017; Hornyák et al., 2018). In this light, it is likely that both pathways could underlie MDD progression and neurotransmitter imbalances that lead to various comorbid symptoms such as mood and behavioral alterations (Mössner et al., 1998; Donegan et al., 2014; Watkins et al., 2014; Figure 3). If a causal relationship between inflammation and MDD stands true, MDD patients may benefit from immunomodulatory treatment and the development of BBB permeable drugs within the IMiD family would become an important therapeutic goal.

### Generalized Anxiety Disorder (GAD)

Anxiety disorders are the most prevalent psychiatric disorder type, affecting around 28.8% of Americans throughout their lifetime, with an estimated 40 million adults experiencing prolonged anxiety. Anxiety disorders can cause not only psychological but also physical symptoms such as chest pain or muscle tension (Sansone and Sansone, 2010). Generalized anxiety disorder treatments include selective serotonin inhibitors (SSRI) (i.e., fluoxetine, escitalopram), anxiolytics, and beta-blockers to treat physical symptoms that follow anxiety attacks. Regrettably, anxiolytic drugs treatment may be accompanied by severe long-term adverse effects such as gastrointestinal, neurological, and cardiac impairments, particularly for patients comorbid with MDD (Shankman et al., 2017). In this light, treatment strategies directed towards different targets that may not adversely interfere with pathways underlying psychiatric disorder and essential homeostatic mechanisms must be investigated.

Treatment of anxiety with anti-inflammatory drugs shows promise in that anxiety symptoms are commonly associated with inflammatory diseases like diabetes and inflammation-induced pain (Felger, 2018; Hu et al., 2019). In preclinical rodent studies of comorbidity, both anxiety and inflammatory symptoms were ameliorated by TNF-α-inhibiting drugs (Chen et al., 2013; Klimov et al., 2018; Fourrier et al., 2019). Similar to MDD, the molecular mechanisms underlying the positive correlation and, perhaps, synergy of anxiety and inflammation are hypothesized to revolve around HPA axis activation and the nervous system response to cortisol-releasing hormone secretion from the paraventricular nucleus of the hypothalamus in response to stress or injury. This activates the locus coeruleus, which ultimately stimulates the sympathetic nervous system and simultaneously deactivates the parasympathetic nervous system. This then leads to elevated levels of norepinephrine and epinephrine and a decrease in acetylcholine, supporting immune cell activation and, in the long run, leading to persistently high proinflammatory cytokine levels in response to prolonged stress (Spengler et al., 1994; Bandelow et al., 2017; Michopoulos et al., 2017). This pathway underlying anxiety could therefore lead to comorbidity of systemic inflammatory diseases with anxiety disorders.

### Post-traumatic Stress Disorder (PTSD)

Within the category of anxiety disorders is post-traumatic stress disorder, or PTSD, which is characterized by intrusive dreams, thoughts, or hallucinations in response to environmental stimuli that represent traumatic events experienced by individuals (Gill et al., 2009). PTSD affects about 8% of the population, and can lead to long-term consequences such as memory impairments associated with hippocampal inflammation (Lee and Yang, 2019). SSRIs are the only drugs currently approved for the treatment of PTSD and provide inadequate treatment, indicating a great need for drug development in this area (Ebenezer et al., 2016).

Post-traumatic stress disorder has been associated with inflammation and a heavy involvement of the immune system (Miller et al., 2017; Speer et al., 2018; Kim et al., 2020). A highly controlled clinical assessment of combat-exposed patients and non-patients of PTSD showed elevated levels of the inflammatory cytokines TNF-α and INF-γ in the incidence of PTSD, as compared to patient controls (Lindqvist et al., 2014). Several studies have also shown upregulation of C-reactive protein, IL-6, TNF-α, and IFN-γ in PTSD patients when compared with healthy controls (Tursich et al., 2014; Cavalcante Passos et al., 2015). This elevation in inflammatory cytokines has been linked to PTSD development in response to traumatic experiences as a result of HPA axis and cortisol regulation failures (Gill et al., 2009; Haroon et al., 2012). Also observed in PTSD is a decrease in GABA, for which low levels can contribute to glutamatergic toxicity and inflammation, revealing another potential reason to target inflammation in such patients (Crowley et al., 2016).

Although there has yet to be evidence of the clinical effects of anti-inflammatory agents in PTSD patients, preclinical studies have shown promising amelioration of cognitive dysfunction by an herbal extract in a PTSD rat model partially through an anti-inflammatory mechanism (Lee and Yang, 2019). Blueberries, which have antioxidant and anti-inflammatory properties, have also been shown to decrease PTSD-associated inflammatory cytokine levels and increase serotonin in a PTSD rat model (Ebenezer et al., 2016). Lastly, there is currently a clinical trial being undertaken at UCSF to investigate the role of chronic and acute inflammation in exaggerated threat sensitivity in individuals with PTSD; this study may ultimately help us to better understand inflammation involvement in PTSD and provide more efficient inflammation targeting strategies (O’Donovan, 2020).

### Bipolar Disorder (BD) and Schizophrenia

Bipolar disorder affects 2% of the global population, ranking 2nd among all health conditions in length of severe individual role impairment (Alonso et al., 2011). Suicide rates of BD patients are 20-30 times higher than that of the general population (Pinto et al., 2017). BD is characterized by manic and depressive episodes, with depressive symptoms presenting at a greater severity than manic symptoms. Anxiety disorder symptoms, especially panic attacks, are the most common comorbid condition (Merikangas et al., 2011). Progressive impairment of cognitive processes, such as attention and executive function, has also been observed in BD patients (Vieta et al., 2013).

Increased levels of systemic inflammatory factors and glial cell activation in the hippocampus have been observed in patients with BD (Kim et al., 2007; Munkholm et al., 2013; Dong and Zhen, 2015). In line with this, biomarkers of astrocyte activation have likewise been described in key brain areas of BD patients (Webster et al., 2005; Pinto et al., 2017). Higher plasma levels of soluble TNF-Receptor 1 (Hope et al., 2009) and proinflammatory cytokines such as IL-6, IL-1β, and TNF-α have also been reported in BD patients compared to controls (Stertz et al., 2013). Moreover, a study of a cohort of BD patients found high comorbidity with metabolic and autoimmune-allergic diseases associated with systemic inflammation, such as diabetes mellitus, psoriasis and irritable bowel syndrome (almost 50%) compared with the general population (Perugi et al., 2015). Schizophrenia patients and BD patients may experience autoimmune diseases prior to disorder onset and similar alterations in inflammatory cytokines (Potvin et al., 2008; Hope et al., 2009; Eaton et al., 2010).

Few clinical trials have tested the effects of anti-inflammatory drugs in BD patients. A meta-analysis on the antidepressant effects of anti-inflammatory agents such as pioglitazone, nonsteroidal anti-inflammatory drugs, and omega-3 polyunsaturated fatty acids tested in randomized trials for bipolar depression showed a significant reduction in depressive symptoms when used in conjunction with antidepressant medication, when compared to conventional therapy without anti-inflammatory agents (Rosenblat et al., 2016). Additionally, a Danish longitudinal study has shown that continued use of low-dose aspirin, statins and angiotensins decreased the rate of incident BD (Kessing et al., 2019), again pointing to the potential repurposing of anti-inflammatory drugs for BD. In a controlled and randomized trial, schizophrenia patients benefitted from treatment with celecoxib, an anti-inflammatory agent (Akhondzadeh et al., 2007).

The role of inflammation in BD progression can be considered to be almost inevitable (Muneer, 2016), as neuronal damage resulting from acute BD episodes can prime microglia to respond to future episodes of excessive proinflammatory cytokine production. This could thereby leave microglia in a consistently activated state (Tay et al., 2018; Benedetti et al., 2020), which coupled with neuronal production of DAMPs and the possibility of peripheral cytokine infiltration, can result in continuous inhibition of neurogenesis (Stertz et al., 2013), potentially leading to further behavioral and cognitive impairments - as described in Figure 3.

If we effectively treat neuropsychiatric disorders highly influenced by inflammation with anti-inflammatory agents, we may be able to reduce the adverse effects of antipsychotics and antidepressant drugs typically used for the treatment of many psychiatric disorders. Taking schizophrenia as a brief example, supplementary treatment of schizophrenia with anti-inflammatory drugs like aspirin, statins, and minocycline has largely proven to be effective in reducing clinical schizophrenic symptoms, as compared to sole treatment with antipsychotics. Schizophrenia patients have been shown to have high levels of inflammatory cytokines, such as TNF-α, IL-1β, and IL-6, as well as high microglial activation throughout the brain as shown by PET scans and postmortem biopsies (Hong and Bang, 2020). Furthermore, there are many forms of evidence suggesting a role for adaptive immunity in schizophrenia patients. Schizophrenia patients appear to have an increased risk for autoimmune disorders such as chronic active hepatitis and thyrotoxicosis, suggesting association of schizophrenia and immune dysregulation (Eaton et al., 2006). NMDA autoantibody encephalitis, which is characterized by brain swelling caused by autoantibody production against NMDA receptors, is a further disorder that provides us with insight into a potential role for immunity in psychosis. Although patients of sporadic schizophrenia do not typically have antibodies against NMDA receptors, NMDA autoantibody encephalitis can present as schizophrenia (Dalmau, 2016), implicating the inflammatory components of either disorder as a potential cause of their psychotic symptoms (Lennox et al., 2017; Chaudhry et al., 2020; Pollak et al., 2014). Other non-neuronal autoimmune disorders, such as psoriasis, have also been reported to be associated with psychosis, with a 45% increased risk for schizophrenia for patients of any autoimmune disease (Eaton et al., 2006; Cullen et al., 2019).

Two recent reviews focused on cytokine imbalances in schizophrenia that detail those that generally become elevated (i.e., TNF-α, IL-1β, IL-6, and IL-12) vs. those that largely are unaltered (e.g., IL-2, IL-4, and IL-17) are by Momtazmanesh et al. (2019), Reale et al. (2021). By and large, there appear to be variable changes in cytokine levels across the different categories of schizophrenia, disease duration and symptom severity, and these may be modified by antipsychotic and/or other treatments. Despite the heterogeneity in data available across studies and the complex association between cytokine levels and clinical status that warrants further clarification, changes in key cytokine levels supports an immunological component in schizophrenia pathogenesis.

In this regard, a recent clinical trial evaluated “low dose” methotrexate plus folic acid in schizophrenia (Chaudhry et al., 2020), as a means to provide an immune-suppressant effect by acting on cell-mediated adaptive immunity with indirect anti-inflammatory actions on the innate immune system. This, in part, mirrors low dose methotrexate’s relatively routine use to treat and reset immune signaling dysfunctions of regulatory T cells in autoimmune disorders, epitomized by rheumatoid arthritis and psoriasis (Friedman and Cronstein, 2019). In the study of low dose methotrexate in schizophrenia (Chaudhry et al., 2020), patients with early schizophrenia spectrum disorders within 5 years of onset were evaluated, as neuroinflammation was considered to still be active and such patients would have less exposure to antipsychotic drugs. Methotrexate treatment provided a selective improvement on positive symptoms in early schizophrenia, without effect on negative symptoms or on cognitive performance. Although the study was not designed to evaluate efficacy, methotrexate proved well tolerated and, largely, exerted an overall improvement in total symptoms and general functioning (Chaudhry et al., 2020). This study, together with others, supports the premise for anti-inflammatory drugs to potentially alleviate psychiatric symptoms or prevent inflammation-related pathways associated with psychiatric disorders. Similarly, several clinical trials have shown anti-inflammatory drugs to improve antidepressant performance in MDD and BD patients, who have similar inflammatory profiles to schizophrenia patients in terms of disorder progression or severity (Husain et al., 2017; Köhler-Forsberg et al., 2019), further supporting the possibility of anti-inflammatory drug use for psychiatric disorder treatment.

### Neurodegenerative Disorders and Neuroinflammation

#### Parkinson’s Disease

Parkinson’s disease is the second most common age-related neurodegenerative disease, affecting 0.1-0.2% of the world’s population (de Lau and Breteler, 2006). PD encompasses both motor and non-motor symptoms. While classical motor symptoms include bradykinesia, resting tremor and rigidity, a number of non-motor symptoms may be prodromal or appear in late stages of the disease. Among them, anosmia, constipation and sleep disorders can appear early and precede motor symptoms (Jankovic, 2008), while depression, anxiety, dementia or mild cognitive impairment/cognitive decline may appear later during the disease course (Barone et al., 2009; Aarsland et al., 2017). Histologically, PD is classically characterized by the degeneration of dopaminergic neurons of the substantia nigra pars compacta (SNc) within the brain and by the presence of Lewy bodies, proteinaceous aggregates enriched in α-Synuclein (α-Syn), in affected areas (Spillantini et al., 1997). Moreover, a neuroinflammatory reaction is consistently reported in the brain of diseased patients, and the pivotal involvement of neuroinflammation in the disease pathogenesis has gained unanimous consensus (Kuter et al., 2020).

Recently, the recognition of the complex motor and non-motor symptomatology and the underlying pathology is changing the view of PD as a SNc-centric disease, in favor of a systemic disease affecting both the CNS and peripheral organs. In line with this multisystem interpretation of the disease and based on the increasing evidence of inflammation not only within the CNS, but also in the blood and peripheral tissues, there is increasing convergence in defining PD as a systemic inflammatory condition (Pajares et al., 2020).

Several studies have demonstrated an imbalance in levels of proinflammatory and anti-inflammatory cytokines and of chemokines in the brain parenchyma and CSF of PD patients. Increased levels of inflammatory cytokines TNF-α, IL-1β, IL-2, IL-6, and IL-4, IFN-γ, but also of the anti-inflammatory cytokine IL-10 and the chemokine CXCL12, have been described to correlate with the clinical course of the disease (Sawada et al., 2006; Mogi et al., 2007; Shimoji et al., 2009; López González et al., 2016; Karpenko et al., 2018). Moreover, epidemiological studies have suggested a link between polymorphisms in the genes encoding for TNF-α and IL-6 and increased risk for developing PD (Krüger et al., 2000). Accordingly, several histological studies in PD patients have reported an overactivation of microglial cells, the main cytokine source in the brain. Microgliosis, characterized by reactive morphology of microglia and the upregulation of inflammatory markers including MHC-II, CD68, ICAM-1, and Toll-like receptors (TLRs), has been observed in many PD patient studies to date (Rozemuller et al., 2000; Imamura et al., 2003; Croisier et al., 2005; Orr et al., 2005; Dzamko et al., 2017). In parallel with these histopathological investigations, PET imaging studies using TSPO showed a chronically increased signal in both subcortical and cortical regions in PD patient brains, signifying chronic elevation of activated microglia (Banati et al., 1997; Ouchi et al., 2005; Gerhard et al., 2006; Bartels et al., 2010; Edison et al., 2013; Iannaccone et al., 2013; Terada et al., 2016). Altogether, these studies suggest that microglia in PD are early and chronically activated in a reactive phenotype, contributing to neurodegeneration via the unremittent release of proinflammatory cytokines (Kuter et al., 2020).

More recent analyses of PD patient serum and peripheral organs has extended the findings of neuroinflammation associated with PD to the whole organism, supporting the concept that PD is a multisystem inflammatory condition (Pajares et al., 2020). Dysregulated cytokine content and higher levels of both proinflammatory and anti-inflammatory cytokines have been reported in the serum of PD patients (Brodacki et al., 2008; Qin et al., 2016). Of note, in a study examining a patient cohort with incident parkinsonism, the unbalanced ratio of increased proinflammatory cytokines versus decreased anti-inflammatory cytokines correlated positively with faster disease progression and cognitive deterioration (Williams-Gray et al., 2016). This study highlighted two important concepts. First, the peripheral inflammatory condition may play a role in the CNS pathology, or at least reflect the course of it, and second, inflammation may be associated with and, perhaps, underlie non-motor symptoms of PD (Williams-Gray et al., 2016). A more recent study questioned whether peripheral immune changes causally contribute to the progression of PD, reporting that serum levels of cytokines do not correlate with CSF content, and suggesting that central and peripheral cytokine levels may partially behave independently, and may be driven by different factors (Wijeyekoon et al., 2020). Additional evidence of a chronic and systemic inflammatory state comes from studies showing the altered profile of immune cell composition in the blood of PD patients as compared to healthy individuals, reporting for instance, an increase in monocyte number and a decrease in the CD4+ T cell to CD8 cytotoxic T cell ratio (Bas et al., 2001; Grozdanov et al., 2014).

As previously noted, constipation is a prodromal symptom of PD (Abbott et al., 2001). Interestingly, an early increase in many proinflammatory cytokines including TNF-α, IFN-γ, IL-6 and IL-1β, has been observed in the gastro-intestinal tract of PD patients (Devos et al., 2013), which has led to the suggestion that gut inflammation may contribute to and even represent an early event in PD pathogenesis (Chen et al., 2019). Of note, a retrospective cohort study of a population diagnosed with inflammatory bowel disease (IBD) revealed a higher incidence of PD among IBD patients compared to healthy subjects, and exposure of these patients to anti-TNF-α therapy was associated with reduced PD incidence (Peter et al., 2018).

At the molecular level, α-Syn is a major component of Lewy bodies and a key player in PD pathogenesis. Diffusible aggregates of α-Syn have been described in damaged areas of PD brains (Sharon et al., 2003; Karpinar et al., 2009) as well as in biological fluids of PD patients (Tokuda et al., 2010; Majbour et al., 2016). α-Syn aggregates have also been observed within the enteric nervous system in the submucosal tissue from the sigmoid colon of PD patients (Beach et al., 2016; Visanji et al., 2017) and enteric α-Syn expression may drive intestinal inflammation (Stolzenberg et al., 2017).

Toxic mechanisms underpinning the damage of α-Syn wrought in the brain are still largely unknown, but α-Syn may impact multiple targets by interacting with neuronal and immune cells. In this light, several findings suggest that α-Syn interaction with microglia is a key event in the neurodegenerative process (Liu C.Y. et al., 2019), driving the shift of these cells to unremittent proinflammatory phenotypes (Villar-Piqué et al., 2016). α-Syn-microglia interaction occurs mainly through TLR2 and TLR4, resulting in NF-κB nuclear translocation and induction of the proinflammatory functions of these cells (Liu C.Y. et al., 2019); leading ultimately to increased production and release of inflammatory cytokines (Stefanova et al., 2011; Fellner et al., 2013; Doorn et al., 2014). Which form of α-Syn is most toxic among intermediates of the aggregation process is still highly debated. However, several studies have shown that the interaction of α-Syn with TLRs is conformation-dependent, and short soluble aggregates such as oligomers display a greater inflammatory and neurotoxic potential than the native monomeric protein (Zhang et al., 2005; Klegeris et al., 2008; Lee E. J. et al., 2010; Rojanathammanee et al., 2011; Fellner et al., 2013; Kim et al., 2013; Daniele et al., 2015; Boi et al., 2020).

Within the enteric nervous system, local inflammation may be triggered by α-Syn to promote systemic and brain inflammation via the production of pro-inflammatory cytokines (Lubomski et al., 2020). Studies aimed at investigating the link between α-Syn and immune responses in PD have suggested a direct involvement of circulating abnormal α-Syn in the dysregulated inflammatory T cell profiles observed in PD, demonstrating the potential for abnormal circulating α-Syn to stimulate cytokine production in peripheral CD4 and CD8 T cells (Sulzer et al., 2017).

Besides its notorious pathological role in the neurodegenerative process, neuroinflammation has recently been under investigation as a possible player in mechanisms underlying the dyskinesia that develops as a consequence of long-term L-DOPA therapy in PD patients (Pisanu et al., 2018). In preclinical studies, an increased production of proinflammatory cytokines such as TNF-α and IL-1β has been associated with a robust activation of glial cells in the DA-denervated striatum of 6-OHDA-infused hemi-parkinsonian rats that developed dyskinesia after L-DOPA treatment (Barnum et al., 2008; Bortolanza et al., 2015; Mulas et al., 2016). Of note, the subcutaneous administration of L-DOPA through osmotic pumps, that maintains a long-term stable plasma concentration of the drug, was not associated with neuroinflammation nor with dyskinetic movements in the 6-OHDA rat model of PD (Mulas et al., 2016). Given the well-characterized neuro-modulatory function of cytokines (Pousset, 1994), and the regulatory action of TNF-α in synaptic plasticity and neuronal excitability (Clark et al., 2010; Clark and Vissel, 2018), it has been suggested that cytokines may contribute to the impairment of cortico-striatal synaptic plasticity that drives the development of abnormal involuntary movements (Carta et al., 2017).

An increasing number of studies strongly indicate that several components of the inflammatory response may represent valuable targets for neuroprotection in PD. Epidemiological and clinical studies have investigated the use of anti-inflammatory NSAIDs in relation to PD incidence, reporting a reduced risk in individuals taking NSAIDs (Chen et al., 2003; Tansey and Goldberg, 2010; Becker et al., 2011). However, more recent meta-analyses have challenged this conclusion (Ren et al., 2018; Poly et al., 2019) or have shown a positive association of PD risk reduction with the use of ibuprofen only (Gao et al., 2011).

In the last decade, clinically available immunosuppressive and immunomodulatory drugs have been successfully tested for their neuroprotective activity in preclinical models of PD (Martinez and Peplow, 2018). Fingolimod and tacrolimus are immunosuppressant agents approved for refractory multiple sclerosis and for prevention of post-transplantation organ rejection, and have been proposed for repositioning in PD following evidence of neuroprotective and anti-inflammatory activity in rodent PD models (Van der Perren et al., 2015; Manocha et al., 2017; Ren et al., 2017; Zhao et al., 2017; Komnig et al., 2018; Motyl et al., 2018). Another immunomodulatory drug, Glatiramer acetate, has a broad effect on cells of both the innate and adaptive immune system. It is used as a first-line agent for the treatment of refractory multiple sclerosis, and has shown neuroprotective properties in 1-methyl-4-phenyl-1,2,3,6-tetrahydropyridine (MPTP) models of PD (Churchill et al., 2019). Unfortunately, despite the promising results of preclinical studies, pharmacokinetic and toxicological caveats limit the clinical translation of these drugs to PD trials. Serious limitations include the systemic adverse effects together with the narrow therapeutic window, and the limited BBB permeability. The immunomodulatory agent Sargramostim (Leukine) has been tested in PD models, displaying a positive effect on circulating regulatory T cell proliferation and neuroprotective and anti-inflammatory effects in the brain (Gendelman et al., 2017). Sargramostim is a human recombinant Granulocyte-macrophage colony-stimulating factor (GM-CSF) that is clinically used for cancer or post-transplantation therapy and is currently in an early-phase clinical investigation for PD (NLM Identifier: NCT03790670).

Other classes of drugs have shown neuroprotection in PD models with mechanisms at least partially involving an inhibitory activity of inflammatory responses. Between them, anti-diabetic drugs are amongst the most promising treatments currently being prioritized for repositioning in these disorders. Oral hypoglycemic glitazones such as pioglitazone and rosiglitazone, which act as peroxisome proliferator receptor (PPAR)-γ agonists, have been proven neuroprotective and anti-inflammatory in a number of different PD models (Escribano et al., 2010; Glass and Saijo, 2010; O’Reilly and Lynch, 2012; Carta, 2013; Papadopoulos et al., 2013; Pisanu et al., 2014; Croasdell et al., 2015; Pinto et al., 2016; Lecca et al., 2018; Machado et al., 2019). Antidiabetic compounds such as sitagliptin, saxagliptin and vildagliptin, which act as glucagon-like peptide (GLP)-1 level enhancers, or exenatide and liraglutide, which are long-acting direct GLP-1 receptor agonists, have shown neuroprotective properties that were at least partially mediated by anti-inflammatory mechanisms in preclinical studies (Kim et al., 2009; Li et al., 2009, 2018; Shiraishi et al., 2012; Abdelsalam and Safar, 2015; Nassar et al., 2015; Badawi et al., 2017). Supporting preclinical results, a recent retrospective study has reported that glitazone use was associated with a significantly lower incidence of PD in diabetic patients (Brakedal et al., 2017), although the single clinical trial testing pioglitazone on PD progression failed to report any improvement in disease symptoms (NINDS Exploratory Trials in Parkinson Disease (NET-PD) FS-ZONE Investigators, 2015). Moreover, A recent clinical trial of exenatide repositioning for PD has produced positive outcomes in motor and cognitive measures, and possibly delayed disease progression (Aviles-Olmos et al., 2014; Athauda et al., 2018).

Raloxifene is a selective estrogen receptor modulator (SERM) prescribed for osteoporosis treatment, and was recently suggested for repurposing in PD following studies showing the neuroprotective and anti-inflammatory activity of this drug in the MPTP mouse model of PD (Bourque et al., 2014; Poirier et al., 2016).

Finally, corticosterone and the PPAR-γ agonist rosiglitazone have shown beneficial effects in parkinsonian rats by attenuating the development of L-DOPA-induced dyskinesia (LID) (Barnum et al., 2008; Martinez et al., 2015). Notably, antiangiogenic compounds such as vandetanib and candesartan also reduce LID in parkinsonian rats, in line with the causal relation linking angiogenesis with neuroinflammation (Ohlin et al., 2011; Muñoz et al., 2014). More recently Exenatide, administered in a sustained-release form that guarantees steady-state plasma levels (PT320), attenuated LID in the 6-OHDA rat model (Yu et al., 2020).

### Alzheimer’s Disease

Parkinson’s disease is the most common form of dementia, affecting more than 40 million of the world’s population (GBD 2016 Neurology Collaborators, 2019). Pathology is characterized by a progressive cognitive decline that usually starts in the form of a Mild Cognitive Impairment (MCI) prior to developing into full-onset dementia. Neuropathology of AD is notoriously characterized by the presence of two main hallmarks: extracellular plaques containing aberrant forms of β amyloid (Aβ), and neurofibrillary tangles (NFT) containing hyperphosphorylated tau protein in the intracellular compartment. These neuropathological hallmarks are accompanied by accelerated atrophy in the brain’s gray matter cortex, such as in the hippocampus and in parietal lobes. Alongside these two core pathologies, in recent years several authors pointed to neuroinflammation as the third characteristic feature of the pathology (Heneka et al., 2014). Some of the first evidences of the involvement of neuroinflammatory processes in the pathogenesis of AD date back to the 80s, when several studies reported the presence of immune-related proteins in the proximity of Aβ plaques (Rogers et al., 1988; Griffin et al., 1989).

Although this relationship was established several years ago, it is still unclear whether neuroinflammatory processes are a cause or a consequence of the disease (VanItallie, 2017). Nevertheless, proinflammatory cytokines seem to play a pivotal role in AD pathology. Both central and systemic signs of inflammation have been observed in AD patients and in animal models of AD. A meta-analysis of 40 studies measuring peripheral blood cytokine concentrations and 14 measuring CSF cytokine concentrations revealed that peripheral cytokines as IL-6, TNF-α, IL-1β, TGF-β, IL-12, and IL-18 are higher in patients with AD (Swardfager et al., 2010). Post-mortem and *in vivo* TSPO measurements of AD brains have shown greater TSPO density in later stages of AD, correlating AD-associated cognitive decline with microglial activation (Edison et al., 2018; Xu et al., 2019). Activated microglia have been consistently observed in post-mortem brain tissue of AD patients (Heneka et al., 2015), further validating the involvement of neuroinflammation throughout AD pathology. Moreover, several genetic factors known to affect AD risk, such as APOE-ε4, PIN1, and BACE1, have been associated with proinflammatory pathways (Sambamurti et al., 2004; Clark and Vissel, 2018; Fernandez et al., 2019). PET imaging of astrocytes using a monoamine oxidase B inhibitor, ^11^C-deuterium-L-deprenyl (^11^C-DED), has shown elevated astrocytosis in early AD and MCI brains as well (Carter et al., 2012), highlighting the potential use of inflammatory biomarkers as a diagnostic tools, and the use of anti-inflammatory or immunomodulating drugs as a therapy for AD.

APP processing and tau phosphorylation, which can lead to Aβ plaque and tau tangle accumulation if dysregulated, are heavily interconnected with inflammatory pathways. Inflammatory cytokines such as TNF and IFN-γ are linked with several signal transduction pathways (Kitazawa et al., 2005; Griffin et al., 2006; Yamamoto et al., 2007; Shaftel et al., 2008). For instance, inflammation activates the cyclin-dependent kinase 5, NF-κB, and mitogen-activated protein kinase (MAPK) pathways, which causes further tau phosphorylation in the hippocampus, a main brain region affected in AD (Kitazawa et al., 2005; Griffin et al., 2006). This strong involvement of cytokines in AD pathogenesis has led to the “damage signals hypothesis” of AD, which postulates that injury or age-related cell stress, via activation of chronic neuroinflammation, is a main cause of neurodegeneration (Maccioni et al., 2009).

Epidemiological studies have examined NSAID use in relation to AD risk (Rich et al., 1995; Chen et al., 2003; Becker et al., 2011)and have suggested slower progression of AD pathology in NSAID users (Stewart et al., 1997; Shankman et al., 2017; Rivers-Auty et al., 2020). In preclinical studies, dimethyl fumarate, an immunomodulatory compound used to treat multiple sclerosis, seems to have neuroprotective effects, reducing neuroinflammation and improving cognitive performance in rats infused with streptozotocin (Majkutewicz et al., 2016). Currently, several immune-oncological and anti-TNF-α compounds are under preclinical and clinical investigation as neuroprotectants in AD, as elegantly reviewed elsewhere in this issue (Munafò et al., 2020). Of note, recent preclinical data have shown that a checkpoint inhibitor against the programmed death-1 (PD-1) protein, clinically used in cancer immunotherapy, resulted in improved clearance of cerebral Aβ plaques and cognitive performance in an animal model of AD (Baruch et al., 2016). The 3 × Tg-AD mouse model shows increased levels of peripheral and CNS inflammatory markers including TNF-related apoptosis inducing ligand (TNFSF10), a potent pro-apoptotic member of the TNF superfamily, which are reverted by treatment with an anti-TNF antibody (Cantarella et al., 2015; Di Benedetto et al., 2019). An increasing number of studies have suggested that modulating inflammation through physical exercise or anti-inflammatory medications is beneficial for preventing AD pathology or mitigating AD symptoms, such as cognitive dysfunction, in various preclinical models of AD (Frankola et al., 2011; Gabbita et al., 2012; Russo et al., 2012; Tweedie et al., 2012; Decourt et al., 2016).

On the other hand, a recent meta-analysis has compared six different antidiabetic compounds for the treatment of AD (i.e., intranasal insulin, pioglitazone, rosiglitazone, metformin, sitagliptin and liraglutide), showing an improvement in cognition in subjects treated with these agents compared with placebo. Notably, among these, pioglitazone demonstrated the greatest efficacy compared with placebo (Cao et al., 2018).

To the present, AD therapeutics have commonly targeted the most well-known hallmarks of the disease - Aβ and tau. However, this approach has failed to produce a drug capable of slowing or preventing AD progression. As a consequence, the currently approved marketed drugs for AD are solely symptomatic. As neuroinflammation seems an early event of AD and is linked to many pathways involved in AD pathogenesis, treating neuroinflammation with TNF-α inhibitors may be a viable way of preventing or slowing AD progression.

## IMiDs and Neurodegenerative Disorders: Preclinical and Clinical Evidence

### Parkinson’s Disease

An increasing number of preclinical studies suggest that IMiDs provide beneficial effects on neurodegeneration in preclinical models of PD. The first evidence of the protective potential of thalidomide was reported by Ferger et al. (2004), who showed thalidomide efficacy in counteracting the MPTP-induced decrease of striatal dopamine (DA). Thalidomide and its analog lenalidomide were tested subsequently in mice overexpressing α-Syn. While these mice developed a deterioration of motor performance associated with loss of dopaminergic striatal fibers, increased cytokine production and microgliosis in the striatum, IMiDs improved all pathological parameters, with lenalidomide being more effective than thalidomide (Valera et al., 2015). Interestingly, IMiDs decreased the α-Syn-induced inflammatory response also in non-motor regions of the brain such as the hippocampus, an area involved in cognitive deficits of PD, supporting a role of neuroinflammation in these non-motor symptoms (Valera et al., 2015; Williams-Gray et al., 2016). A recent study demonstrated the pomalidomide efficacy in a drosophila LRRK2 WD40 mutant PD model, with LRRK2 being a common genetic cause of PD (Casu et al., 2020; Dues and Moore, 2020). These flies develop motor impairment and gradually lose dopaminergic neurons with age (Casu et al., 2020). Dietary administration of pomalidomide prevented age-dependent motor impairment and neuronal loss in motor-related dopaminergic clusters (Casu et al., 2020). The pomalidomide derivative 3,6′-DP has also been shown to reduce cell loss in primary dopaminergic neuron cultures exposed to α-Syn oligomers (Lin et al., 2020). Additional studies are warranted to further test the efficacy of IMiDs in mammalian models of PD neuropathology, to investigate potential use for non-motor symptoms of PD, and to understand the systemic drug effect on inflammatory markers and immune cell activation in PD models.

Immunomodulatory imide drugs have also been tested for effectiveness against LID in a preclinical PD model, as LID is acommon complication following dopamine-replacement therapy in PD. Thalidomide and its more potent analog, 3,6-DTT, significantly attenuated the severity of LID in a rat model of PD (Boi et al., 2019). This effect was associated with a reduction of L-DOPA-induced striatal inflammatory cytokines, including TNF-α, and with the restoration of pro-inflammatory/anti-inflammatory cytokines ratio (Boi et al., 2019). A normalization of the L-DOPA-induced expression of AMPA receptor subunit GluR1, which is modulated by TNF-α levels and contributes to the synaptic abnormalities underlying dyskinesia, was also observed in this study (Boi et al., 2019; Konitsiotis et al., 2000). Furthermore, thalidomide was able to inhibit the angiogenesis characteristic of LID, in accord with the potent antiangiogenic activity of this drug (Boi et al., 2019), and with the antidyskinetic properties of anti-angiogenic compounds (Ohlin et al., 2011; Kuter et al., 2020).

### Alzheimer Disease

The neuroprotective role of IMiDs has been evaluated in different preclinical models of AD. In this regard, in two different studies, Elçioglu et al. (2013), Kübra Elçioğlu et al. (2015) observed that thalidomide was able to provide neuroprotection from memory deficits and neuronal damage induced by intracerebroventricular (ICV) infusion of streptozotocin. Moreover, the anti-inflammatory properties of thalidomide have been evaluated in Aβ1-42 peptide-infused rats, where a reduction of both microgliosis and astrogliosis in the hippocampus was observed after thalidomide administration (Ryu and McLarnon, 2008).

Thalidomide derivatives have been tested in both *in vitro* and *in vivo* models of AD. Tweedie and colleagues have evaluated the efficacy of different thalidomide-derivatives, including several newly characterized compounds, in an *in vitro* mouse macrophage-like cellular screen, where RAW 264.7 cells were exposed to lipopolysaccharide (LPS) in order to induce a rapid concentration-dependent cellular release of TNF-α (Tweedie et al., 2009). The authors observed that some derivatives, such as 3,6′-dithiothalidomide (3,6′-DTT), dithioglutarimide and dithiopthalimide, displayed a more potent TNF-α lowering activity when compared with thalidomide (Tweedie et al., 2009). These results have been confirmed *in vivo* by Gabbita et al. (2012), Tweedie et al. (2012), who evaluated the effects of thalidomide and 3,6′-DTT in the 3 × Tg-AD mouse model. In fact, a significant decrease in TNF-α levels was observed in animals treated with 3,6′-DTT, whilst a milder effect was observed in the thalidomide-treated group (Gabbita et al., 2012). Moreover, even though both agents were effective at reducing the total number of microglial cells, only 3,6′-DTT increased the ratio of resting to activated microglia, resulting in a morphological profile of microglia in the hippocampus similar to Non-Tg mice (Gabbita et al., 2012), and only 3,6′-DTT mitigated cognitive impairments (Gabbita et al., 2012; Tweedie et al., 2012). Likewise, dithiopthalimide (Zhu et al., 2003; Tweedie et al., 2009) has demonstrated the ability to mitigate markers of neuroinflammation and lowered tau and amyloid accumulation as well as cognitive deficits in a 3 × Tg-AD mouse model – as evaluated under the compound name of isoindolin-1,3 dithione (Gabbita et al., 2015), without reference to its original synthesis (Zhu et al., 2003).

In view of this promising evidence, a clinical trial was recently designed to assess the tolerability and the beneficial effects, in terms of cognitive symptoms, of escalating doses of thalidomide in a cohort of mild to moderate AD patients (Decourt et al., 2017). Unfortunately, many patients reported very poor tolerability for thalidomide at the dose selected to provide anti-inflammation, and terminated the study early or did not reach the therapeutic dose. Without reaching the predefined efficacious dose, not surprisingly there were no beneficial effects on cognition, leading to the failure of the study (Decourt et al., 2017). A clinical trial is currently ongoing to evaluate the beneficial effect of Lenalidomide in patients with Mild Cognitive Impairment and AD (Decourt et al., 2020).

#### Acute Neurodegenerative Disorders – Traumatic Brain Injury and Ischemic Stroke

Traumatic brain injury represents a major cause of death and long-term disability in the developed world, with an excess of 10 million people suffering such injury worldwide annually (Hyder et al., 2007). Although the vast majority of TBIs are mild to moderate and account for 80–95% of cases, with severe TBI comprising the remains (LoBue et al., 2019), recovery is generally incomplete and significant and lifelong cognitive, physical, and behavioral deficiencies routinely occur and require long-term access to health care and disability services (LoBue et al., 2019; Pavlovic et al., 2019). It is now recognized that TBI represents a time-dependent process activated at the instance of injury, rather than a single event, and whether clinically manifested or asymptomatic, TBI is one of the most powerful environmental risk factors that leads to the later development of dementia; in particular PD and AD (Barnes et al., 2014; Gardner and Yaffe, 2015; LoBue et al., 2019). A further major risk factor leading all causes of dementia is ischemic stroke (Kuźma et al., 2018), the second leading cause of death worldwide (Donkor, 2018).

It is widely recognized that inflammatory cytokines and chemokines play significant roles in the pathophysiology of both TBI and stroke. Although initiation of an inflammatory response can be indispensable to initiate reparative processes in brain following a physiological challenge, should this be unregulated and excessive, inflammation can drive neuronal dysfunction and degeneration by inducing a self-propagating pathological cycle (Morganti-Kossmann et al., 2002; Frank-Cannon et al., 2009; Frankola et al., 2011). Minutes after TBI or stroke, extensive generation and liberation of proinflammatory cytokines ensues from microglia and astrocytes. In particular, TNF-α protein and mRNA levels rise remarkably acutely, preceding the appearance of ensuing cytokines, and leading to their induction and release (Shohami et al., 1997; Frugier et al., 2010; Yoon et al., 2013; Tuttolomondo et al., 2014; Baratz et al., 2015).

Lowering but, nevertheless, retaining the early-phase release of TNF-α following a TBI or stroke has been achieved in preclinical animal models of mild and moderate TBI, and stroke by thalidomide analogs. Specifically, 3,6′-DTT mitigated TBI-induced cognitive impairments when administered up to 12 h post-mild concussive injury, and reduced neuronal cell loss and apoptosis, glial cell activation and brain TNF-α levels (Baratz et al., 2011, 2015). 3,6′-DTT, likewise, in a rat model of moderate to severe (controlled cortical impact (CCI)) TBI reduced the cortical contusion volume and number of apoptotic neurons associated with it, mitigated microglial activation and lowered protein and mRNA levels of TNF-α as well as IL-1β and IL-6, lowered markers of oxidative stress and mitigated TBI-induced behavioral impairments (Batsaikhan et al., 2019). 3,6′-DP has similarly been evaluated in a rat CCI model of TBI, and compared to equimolar pomalidomide (Wang et al., 2016; Lin et al., 2020). Both demonstrated efficacy, reducing the TBI-induced contusion volume and multiple key markers of neuronal cell death, microglial and glial cell activation, cytokine levels and behavioral impairments, but with 3,6′-DP proving to be approximately 5-fold more potent than pomalidomide (Lin et al., 2020). Finally, NAP has been evaluated in a mouse CCI TBI model and, similarly, proved efficacious by mitigating neuronal and synaptic loss, neuroinflammation and behavioral deficits (Hsueh et al., 2021).

With regard to ischemic stroke, 3,6′-DTT and thalidomide have been evaluated, side-by-side, in the classical middle cerebral artery occlusion/reperfusion (MCAO/R) mouse stroke model (Yoon et al., 2013). 3,6’-DTT administration following stroke reduced infarct volume, neuronal cell death and neurological deficits, whereas thalidomide was effective only when administered prior to stroke induction. 3,6′-DTT neuroprotection was accompanied by decreased inflammation, reduced TNF-α and IL-1β brain levels, decreased microglial and astrocyte activation, and attenuation of BBB disruption. Notably, treatment with 3,6’-DTT did not decrease ischemic brain damage in mice lacking TNF receptors (Yoon et al., 2013). This is consistent with a critical role for suppression of TNF-α production and signaling in the therapeutic action of 3,6’-DTT and related IMiDs, and suggests that anti-inflammatory mechanisms largely underlie the therapeutic actions of this drug class.

Finally, thalidomide has been evaluated in a rodent traumatic spinal cord injury (SCI) model, when combined with rolipram - a phosphodiesterase type 4 inhibitor that was developed as an antidepressant, but discontinued due to its narrow therapeutic window. This SCI model reproduces the elevated TNF-α and IL-1β generation and release evident in the previously described TBI and stoke models, and alike human conditions (Koopmans et al., 2009). The combination of a high thalidomide dose and rolipram attenuated TNF-α and IL-1β levels, augmented white matter sparing at the lesion epicenter and, thereby, improved behavioral outcome (Koopmans et al., 2009). Thalidomide alone, however, failed to provide efficacy (Koopmans et al., 2009; Reyes-Alva et al., 2009), except when administered as a particularly high dose in a mouse compression injury study involving a slightly different form of trauma (Genovese et al., 2008).

In closure, both 3,6′-DTT and 3,6′-DP have demonstrated efficacy in a bilirubin toxicity-induced mouse model of hearing loss (Schiavon et al., 2018). In line with hyperbilirubinemia (jaundice) that can occur in premature newborns and lead to brain damage and/or hearing loss (Shapiro et al., 2006; Haustein et al., 2010), the exposure of mice to an acute high bilirubin dose resulted in hearing loss, ataxia and kernicterus via mechanisms that encompassed neuroinflammation, NF-κB activation, endoplasmic reticulum (ER) stress and activation of the unfolded protein response (UPR) (Schiavon et al., 2018). Interestingly, there was considerable overlap between the bilirubin-induced toxicity in the auditory pathway with hallmarks induced by exposure to LPS – the well-characterized inducer of neuroinflammation – and hearing loss was mitigated by both 3,6′-DTT and 3,6′-DP (Schiavon et al., 2018).

## Conclusion

As a rule, the development and use of animal models of complex human diseases is valuable for studying the biological bases of these disorders and for identifying new drug targets to provide more effective future treatment. Preclinical models potentially permit the evaluation and monitoring of disease progression more rapidly than is feasible in humans, and allow invasive studies to characterize molecular, biochemical and structural changes during disease progression to, thereby, support testing of new therapeutic strategies and potential drugs. Certainly, the human disease is invariably far more complicated than the animal model. However, for neurological disorders, the complexity of the human brain, as compared to that of a rodent or even of a non-human primate, is extreme. For neuropsychiatric disorders, such as schizophrenia, common symptoms such as paranoid delusions and auditory hallucinations are uniquely human and make interpretation of results acquired from animal models particularly challenging (Canetta and Kellendonk, 2018). Although imperfect, there are multiple available models of schizophrenia (Winship et al., 2019), depression (Planchez et al., 2019), and other neuropsychiatric (Nestler and Hyman, 2010) as well as neurodegenerative disorders (Dawson et al., 2018; Carta et al., 2020) that, to varying degrees, possess face validity (observed characteristics/symptoms that have clinical correlates in the human patient population), construct validity (key neurobiological/pathological bases of the human disorder), and/or predictive validity (expected pharmacological response to efficacious drugs currently used to treat the human disorder). Each animal model has its advantages and caveats (Fisher and Bannerman, 2019), and evaluation of hypotheses as well as potential drugs across models undoubtedly provides better predictive value for translation to human studies, particularly when repurposed drugs (such as IMiDs) are evaluated at a clinically translatable dose (Becker and Greig, 2010; Seeman et al., 2019).

To date, IMiDs have largely been evaluated in animal models of neurodegenerative disorders (AD, PD, TBI, SCI, ischemic stroke, and hyperbilirubinemia-induced neural damage), although some have been evaluated in models of multiple sclerosis (Contino-Pépin et al., 2009; do Amaral Corrêa et al., 2010; Karlik et al., 2012; Eitan et al., 2015) that, too, possess aspects of neurodegeneration. These IMiD studies have shown consistent signals of biochemical and immunohistochemical efficacy, and improved behavioral outcomes. Second, third and later generation agents have demonstrated improved efficacy over the first generation drug, thalidomide, which is the only agent for which human clinical trial data in neurodegenerative disorders is currently available and, as noted, is compromised by adverse, dose-limiting actions prior to reaching and maintaining a predicted anti-neuroinflammatory dose. The most promising IMiDs from these preclinical neurodegenerative disorder studies should now be evaluated in animal models of schizophrenia, depression and other neuropsychiatric disorders in which a neuroinflammatory component has been documented.

## Future Studies

Normalizing levels of proinflammatory cytokines appears to provide a rational approach to effectively treat inflammatory aspects of neurological disorders. Key among the targets is TNF-α as a master regulator of the inflammatory process, and whose targeting in the treatment of rheumatoid arthritis and a broad number of autoimmune disorders has provided a huge improvement in the management of these illness (Feldmann et al., 1998, 2010; Feldmann and Maini, 2008; Shepard et al., 2017). Anti-TNF-α monoclonal antibodies are among the most widely used medicines worldwide (Pharmaceutical Technology, 2019; Urquhart, 2020). Unfortunately, these biological drugs are not best suited for treating neurological disorders due to their limited BBB permeability. In this context IMiDs, with their ability to effectively decrease TNF-α and, in general, proinflammatory cytokines levels provide a promising alternative option for the treatment of neurological disorders. IMiDs have shown beneficial effects on neurodegenerative disorders by targeting inflammation. Several of the early generation, FDA approved IMiDs, including thalidomide, lenalidomide and pomalidomide, have been tested in pre-clinical studies as potential therapies for neurological conditions such as AD and PD. Preclinical studies have suggested that therapeutic effects of IMiDs may cover both motor symptoms in PD and cognitive symptoms in both AD and PD. Clinical trials for testing IMiDs in PD have not been designed yet, while one clinical trials in AD patients is in progress (Jung et al., 2019; Decourt et al., 2020). Moreover, the first clinical trial in AD patients was terminated prematurely due to drug toxicity and tolerability issues (Decourt et al., 2017), indicating a need for the development of more potent and less toxic IMiDs. In particular, research of more potent IMiDs against TNF-α synthesis, and for agents that do not racemize from R to S-enantiomers and/or do not bind to CRBN would yield important results in the search of novel neuroprotective immunomodulatory drugs. Although IMiDs have yet to be tested in other neurological conditions, such as psychiatric disorders and MDD, it is quite possible that this drug class would ameliorate neurological disorder phenotypes associated with an inflammatory setting. There is substantial evidence that such an inflammatory microenvironment is associated with both neuropsychiatric and neurodegenerative disorders, and hence the application of IMiDs to this critical and unmet area of medicine provides a treatment avenue worthy of exploration. Who to treat; when to treat; how to best treat; which drug(s) to evaluate and which markers of response to follow are critical questions to now carefully consider?

## Author Contributions

YJ did the concept, literature search, initial draft, and editing. DT, MS, DK, MP, and AP performed the literature search, secondary writing or intellectual input, and editing. AC and NG did the concept, literature search, primary writing, and editing. All authors contributed to the article and approved the submitted version.

## Conflict of Interest

DT and NG are named inventors on patents covering novel thalidomide analogs and have assigned all their rights to the National Institute on Aging, National Institutes of Health. DK was supported by the AevisBio (Gaithersburg, MD, United States and Daejeon, South Korea), a company with a research and development interest in treating neurodegenerative disorders, and an approved Cooperative Research and Development Agreement with NIA, NIH. The remaining authors declare that the research was conducted in the absence of any commercial or financial relationships that could be construed as a potential conflict of interest.
